# Our Contemporaries

**Published:** 1916-02

**Authors:** 


### OUR CONTEMPORARIES

You want a larger and better Journal

YOU CAN HAVE IT BY WRITING OUR ADVERTISERS: "I SAW YOUR AD IN OUR STATE JOURNAL."

FAVOR THOSE WHO FAVOR US.

Reproduced from New Mexico Medical Journal, the organ of the state society. There is no east, no west, no north, no south so far as this truth is concerned. It is not even limited to medical journals.

The Buffalo Express celebrated its 70th anniversary January 15, issuing a fac-simile of the first issue, which was surprisingly large and interesting for a new venture. This Journal is, we believe, the only extant periodical of any kind in Western New York that antedates the Express. We extend our hearty congratulations and best wishes.

The American Journal of Orthopaedic Surgery, with the January issue of this year, changed its place of publication from Philadelphia to Boston, and will begin Vol. 14 as a monthly instead of quarterly. Dr. Mark Rogers is now the editor. It will continue to be the official organ of the Am. Orthopaedic Assn. but will not confine itself to the transactions of this association. It is said to be the only journal in English devoted entirely to bone and joint surgery. The publisher is Ernest Gregory. Our best wishes to the Journal in its expansion.

Eugenical News is a new publication, appearing as a small bi-monthly bulletin, from the Eugenics Record office at Cold Spring Harbor, L. I.

We were wrongly informed as to the purchase of the Medical Review of Reviews. This journal will continue as an independent publication, under the editorship of Victor Robinson. Dr. Wm. J. Robinson's connection with the Review of Reviews, except as a contributor and co-operator, covered the years 1910-11; then it passed into the hands of Frederic H. Robinson, who combined Therapeutic Medicine with it. The January issue, beginning Vol. 22, opens with A Review of the Medical Review of Reviews, being the New Editor's Salutatory, gives an interesting history of the evolution of the journal from its beginning. Frederic H. Robinson is the publisher of the Journal.

Dr. Ira S. Weil, formerly editor of the Medical Review of Reviews, has been appointed to the editorial staff of American Medicine.

The Journal of Cancer Research is to be issued quarterly, beginning with January, 1916. This new journal will be the official organ of the American Assn. for Cancer Research, and will be edited by Dr. Richard Weil of New York City, assisted by a committee of the Assn.

**Dwarf Small-pox.**—The usually sane Buffalo Medical Journal discusses "The Possibility of Vaccination Against the Exanthemata." The nub is, that as vaccination sets up a "dwarf" small-pox, which is not so fatal as the big small-pox, why not give the people all the other diseases in this category in the same manner? This is badly put by us, but it is the gist of the argument. Are not these "dwarf" diseases inflicted on all but a return to the worship of the Hindoo goddess of disease, namely, to voluntarily become ill to appease her? Isn't health better than a "dwarf" disease? Is it necessary to depart from health to retain health?—Homoeopathic Recorder, Jan.

